# Effects of concentrate main carbohydrate type on methane emissions, milk production and feeding behavior in grazing late-lactation dairy cows

**DOI:** 10.3389/fvets.2026.1856960

**Published:** 2026-06-19

**Authors:** Maria M. Della Rosa, Troy J. Bosher, Muhammad A. Khan, Norton Atkins, Arjan Jonker

**Affiliations:** 1 Grasslands Research Centre, Bioeconomy Science Institute, Palmerston North, New Zealand; 2 School of Agriculture and Environment, Massey University, Palmerston North, New Zealand; 3 Agriculture and Horticulture Enterprises, Massey University, Palmerston North, New Zealand

**Keywords:** carbohydrate type, compound feed, greenhouse gas, *Lolium perenne*, Perennial ryegrass

## Abstract

Dairy production in temperate regions relies on herbage as the main source of feed and supplements are fed to cope with a decrease in herbage quality and quantity during summer. The objective of the current study was to evaluate the effect of no supplementation or supplementation with high-starch, high-fiber, or mixed concentrates (50 starch:50 fiber) in grazing late lactation dairy cows on methane (CH_4_) emissions, milk production and feeding behavior. Seventy-two multiparous lactating dairy cows (Holstein-Friesian × Jersey; 206 ± 9.5 days in milk) were assigned to receive 0 kg/d of concentrate (CTR) or 5 kg/d of high starch (STA), high fiber (FIB) or a 50:50 mix of STA and FIB concentrate (MIX) during milking. Methane production was measured using two GreenFeed units for 36 days, with 60 cows having sufficient visits to be included in the data analysis. Feeding behavior was recorded using Nedap CowControl™ collars. Milk production was recorded daily, and milk composition was determined weekly. The daily CH_4_ production was similar among dietary treatments. The fat and protein-corrected milk production (FPCM) was 16% greater in MIX and STA compared to CTR cows (*P* < 0.01), with FIB cows intermediate. Methane as g/kg FPCM was 14 and 13% less (*P* = 0.02) in MIX and STA compared to CTR-fed cows, with FIB cows intermediate. Eating rate and rumination rate (min/kg of dry matter intake) were positively correlated with CH_4_ expressed as g/kg FPCM and as g/kg of total solids (*r* = 0.30 to 0.44). In conclusion, supplementing grazing late lactation dairy cows in summer with concentrates can be a strategy to increase FPCM and reduce the CH_4_/FPCM.

## 1 Introduction

Livestock are responsible for about 43% of global anthropogenic methane (CH_4_) emissions, with cattle alone accounting for 64%−78% of the CH_4_ generated within the livestock sector (1). Methane is formed during the fermentation of feed in the rumen and the dry matter intake is the main driver of CH_4_ emissions in ruminants (2). Dairy production is predominantly pasture-based in temperate regions, such as New Zealand, and some parts of Australia, South America, the United States, and Europe. Cows in these systems are mainly maintained on a fresh herbage diet, making pasture-based systems the cornerstone of dairy production (3).

Perennial ryegrass (*Lolium perenne*) is the most widely sown grass species in temperate regions (4). Ryegrass provides high-quality herbage in spring and autumn, but the nutritive value and availability of ryegrass herbage decline in summer (5), while the nutritional requirements, body condition score gain, and pregnancy of late lactation cow remain relatively high. Supplements are used to support or increase milk production in response to these seasonal herbage gaps (6). The magnitude of the milk production response to supplementation and CH_4_ production per kg of milk depend on the characteristics of the supplement and the substitution rate of the basal herbage diet (6).

Supplements used in dairy farming include those rich in starch, such as corn and barley grain, and those rich in fiber, such as food industry by-products. The choice of concentrate used depends on the cost, availability and/or quality of the main component of the diet (7). The supplementation of high starch concentrates is generally used to provide more energy to the animals than the energy provided by herbage alone. Conversely, the high fiber supplementation is provided mainly to mitigate the seasonal pasture shortage. Increasing the energy provided by the diet or the dry matter intake by supplementing high starch and high fiber concentrates, respectively, are likely to increase the net CH_4_ production (g/d) (8–10). However, the CH_4_ production per unit of milk produced would decrease as a consequence of greater milk production (6).

Changes in diet quality and form also affect the feeding behavior. Decreasing the forage-to-concentrate ratio has been shown to decrease the time dedicated to harvesting of herbage and ruminating ingesta (11). This change to the diet's components directly affects the eating rate (g dry matter intake/min) and rumination rate (g dry matter intake/min), which have previously been associated with changes in CH_4_ production. Biswas et al. (12) found that shorter meal sessions were associated with lower CH_4_ yield (g/kg dry matter intake), possibly due to a lower ruminal pH reached by faster eaters. Rumination is described as a complementary digestive activity that helps to buffer pH drops (11), and increases in rumination time could be associated with increases in CH_4_ yield.

The effect of supplements on CH_4_ and milk production from grazing cows depends on the quality and quantity of the base diet (13). It was hypothesized that the supplementation with high starch and mixed (50:50 starch: fiber) concentrates would increase milk production in late lactation dairy cows grazing ryegrass-based herbage in summer and consequently decrease CH_4_ per unit of milk compared to non-supplemented cows and high fiber supplemented cows. Furthermore, it was hypothesized that the supplemented cows would have a higher eating rate and spend less time ruminating. The objective of the current study was to determine the effect of supplementing late-lactation grazing dairy cows with high starch, high fiber or mixed concentrates on CH_4_ emissions, milk production and feeding behavior.

## 2 Materials and methods

This dairy cow study was approved by the AgResearch Animal Ethics Committee (AE approval #717), and animal use adhered to the guidelines of the 1999 New Zealand Animal Welfare Act and the AgResearch Code of Ethical Conduct.

### 2.1 General management and dietary treatments

The experiment determined CH_4_ emissions using GreenFeed monitoring units (GF, C-Lock, South Dakota, USA) from 72 late-lactation dairy cows (Holstein-Friesian × Jersey; 3–6 years old) fed different types of concentrates and grazing perennial ryegrass-based herbage at Dairy farm No. 4 (Massey University, Palmerston North, New Zealand, S 40° 23′ 49″, E 175° 36′ 46″). The cows were selected from a pastoral spring calving herd, initially for an early lactation spring study described by Bosher et al. (10) that finished on 18th of November 2022. In that study, the cows received graded levels of concentrates. The current study started on 6th of February 2023 and ended on 5th of April 2023. For health reasons, six cows from the previous study were replaced with new cows from the same spring calving herd. The 72 cows were 206 ± 9.5 (average ± standard deviation) days in milk, had a body condition score (BCS) of at least 4 (on a 10-point scale, where 4 is the minimum of BCS at planned start of mating) and a body weight of 531 ± 50 kg before the beginning of the study. The cows had a breeding worth index of 183 ± 59 and a production worth index of 196 ± 122 (14). The cows were 4.1 ± 0.52 months pregnant, except for seven cows that were not pregnant for the duration of this study. The 72 cows (4.7 ± 1.1 years old) were blocked by parity number (parity two, *n* = 24; parity three, *n* = 27; and parities four to five, *n* = 21) and treatment group from the previous study [0, 2, 4, and 6 kg concentrates/cow/d (10)]. The blocking strategy was used for treatment assignment to prevent potential biases related to previous dietary treatments applied by Bosher et al. (10). In addition, more than 60 days elapsed between the end of one experiment and the start of the next, during which cows were fed a basal farm diet to wash out any carryover effects.

The cows within each of the 12 blocks were randomly assigned to one of the four dietary treatments (*n* = 18 per treatment): on a dry matter (DM) basis, 0 kg/d of concentrate (CTR), 5 kg/d of high starch concentrate (STA), 5 kg/d of high fiber concentrate (FIB), and 2.5 kg/d of STA plus 2.5 kg/d of FIB (MIX). After this random allocation, it was checked that the average and standard deviation (SD) of pre-study body weight (BW), breeding worth index and production worth index (and number of empty cows) were similar across treatment groups. The STA pellets were composed of 10% corn grain, 13% wheat grain, 30% barley grain, 30% brollard, 7.5% palm kernel expeller, 5% molasses and 4.5% of soybean meal (Denver Stock Feeds Ltd., Palmerston North, New Zealand). The FIB concentrate pellets comprised 30% brollard, 40% palm kernel expeller, 5% sugarcane molasses, 23% soybean hulls and 2% soybean meal (Denver Stock Feeds Ltd.).

The cows were gradually transitioned from their farm diet to their respective treatment diet across 14 days. The farm diet consisted of grazed ryegrass-based herbage supplemented with 2 kg DM of maize silage, 4 kg DM of perennial ryegrass baleage, 1 kg DM of sugarcane molasses, 1 kg DM of soybean hulls and 0.055 g urea. After the diet transition, the cows were adapted for another 14 days on their respective full-treatment diet before the start of the measurement phase of 36 days.

The cows were milked twice daily at approximately 06:30 and 13:30 h in a 28-bale rotary milking shed with an in-shed feeding system and electronic ear tag reader (Waikato Milking Systems, Hamilton, New Zealand). The in-shed feeder in the milking shed is connected to two feed silos via piping with augers. The feed delivery of each concentrate feed (STA and FIB) was calibrated and tested weekly to ensure that the amount of pellets delivered was accurate and consistent over time. The concentrates were fed in two allocations (50% of the feed each) during the morning and afternoon milking. Refusals were visually monitored after the cows left the bale and were collected if present. The dry matter intake (DMI) of concentrates was calculated as DM offered minus DM refused. Each milking lasted 45–60 min and after each morning milking the cows were offered a new strip of ryegrass-based pasture (no back fencing). The size of the strip was calculated to offer on average 22 kg of DM of pasture per cow (pasture mass of ~3,000 kg DM/ha), leaving a residue of at least 1,500 kg DM/ha after grazing. The cows were also offered 2 kg DM perennial ryegrass silage mixed with 322 g minerals per cow fed in the paddock every morning (after milking). The mineral mix was composed of 1.6% microminerals mix (AquaTrace 5, Nutritech, Auckland, New Zealand), 12.3% urea, 4.8% zinc oxide (Global Supa Zinc, Interchem, Auckland, New Zealand), 63.0% calcium carbonate (Lime Flour, Ravensdown, Christchurch, New Zealand), 6.1% magnesium oxide (Magnesium Oxide Fine, Ravensdown) and 12.3% sodium chloride (Salt, Ravensdown).

### 2.2 Methane measurements using the GreenFeed units

The measurements of CH_4_ and carbon dioxide (CO_2_) emissions were carried out over 36 days using two GreenFeed (GF) automated emission monitoring units (Unit #76 and Unit #77; C-Lock Inc.). The GF units were generator-powered and trailer-mounted (15). The gas analyzers of the GF units were calibrated before the measurement phase and weekly during the measurement phase. The span gas mixture used for calibrating the sensors of unit 76 contained 1,002 ppm CH_4_ and 10,000 ppm CO_2_ (BOC, Linde, Auckland, New Zealand) and the span gas for unit 77 contained 500 ppm CH_4_ and 5,000 ppm CO_2_ (Coregas, Auckland, New Zealand). A CO_2_ recovery test was performed before starting the measurement phase, in the middle and at the end of the measurement phase. The measurements were preceded by a 2-week training/adaptation of cows to use the GF units and during this training phase, the GF units were set up without chutes or any other restrictions to use the GF units. During the measurements phase, cow access to the GF was managed by a chute (raceway) of ~2 m long and 0.85 m wide. During the measurement phase, 32 cows left the herd for approximately 48 h in eight groups of four cows for respiration chamber measurements (16) (covered from ~08:00 h on calendar day 1 to 08:00 h on calendar day 3, information used to process the behavioral data). After the groups of four cows came back to the farm, at around 09:00 h, they immediately rejoined the rest of the study herd.

The GF units were moved daily to the new strip in the paddock to keep the GF in close proximity to the cows. The cows were attracted to visit the GF units using lucerne-based pellets (Alpaca and Llama Pellets, Country Harvest, Hamilton, New Zealand). The units were both set to release seven drops of pellets per visit at 25-s intervals into a covered feed dish. A waiting time of 2 h was set before the same cow could receive pellets from the GF units again. The amount of pellets released per feed drop was, on average 34 ± 1.3 g (as is), which was based on the weekly weighing of 10 feed drops. A sample of pellets was collected weekly for DM determination and chemical composition analysis (see below). Methane and CO_2_ emissions (g/d) for each visit to the GF units that lasted 1.5 min or longer were supplied in a spreadsheet by C-Lock.

### 2.3 Feed sample collection and analyses

Pre-grazing herbage samples were collected three times weekly during the measurement phase on Mondays, Wednesdays, and Fridays. A composite herbage sample for each grazing date was collected by cutting herbage to 5–7 cm above ground level. Approximately 12 subsamples of ~70 g of fresh material were collected, randomly distributed across the strips to be grazed until the next herbage sampling day. A subsample of each pasture sample was oven-dried at 105 °C for 24 h (in triplicate) for DM determination and disposed of. A second subsample was frozen at −20 °C for later chemical analysis. Once a week, a third subsample of pasture was also collected to determine the botanical composition of the herbage. The species identification was performed in triplicate, for a total of five samples. The pasture across the study's grazing area contained on average (on a DM basis) 76 ± 16% perennial ryegrass (*Lolium perenne*), 12 ± 10% white clover (*Trifolium repens*), 7 ± 11% chicory (*Cichorium intybus*), 1 ± 2% red clover (*Trifolium pratense*) and 4 ± 9% weeds. Samples of STA, FIB and lucerne pellets were collected once a week and were stored at room temperature till subsequent chemical analyses. Herbage silage samples were collected twice a week, after the minerals had been mixed in the silage. A weekly subsample of each feedstuff was oven-dried at 105 °C for 24 h (in triplicate) for DM determination, and a second subsample of herbage silage was frozen at −20 °C for later chemical analysis.

All frozen herbage and herbage silage sub-samples were freeze-dried and ground to pass a 1 mm screen before chemical analyses. Individual herbage samples were analyzed using near infrared spectroscopy (FT-NIR Multi Purpose Analyzer, Bruker MPA, Ettlingen, Germany) by the Nutrition Laboratory at Massey University (Palmerston North, New Zealand) for ash, crude protein (CP), neutral detergent fiber (NDF), acid detergent fiber (ADF) and lipids as described by Corson et al. (17). Calibration equations for near-infrared spectroscopy were developed using over 800 herbage samples, primarily composed of ryegrass herbage, whose chemical composition had been determined through wet chemistry analyses, to enable the prediction of herbage composition. Pooled herbage samples (*n* = three pools) were analyzed for mineral composition at Hill Laboratories (Hamilton, New Zealand) as described below. The metabolizable energy for fresh herbage was estimated using Eq.1.14 detailed in CSIRO (18).

Herbage silage samples were pooled (*n* = four pools), as well as FIB, STA, and lucerne pellet samples (*n* = two pools per feed type), before chemical composition analyses. The wet chemical composition for pooled samples of STA, FIB and lucerne pellets and herbage silage was analyzed by Hill Laboratories (Hamilton, New Zealand). Samples were analyzed for ash (AOAC method 942.05), crude protein (*N* × 6.25; AOAC method 968.06), and starch (AOAC method 996.11) content following the AOAC (19) procedures. Crude fat was extracted using petroleum ether in an ANKOM XT15 Extractor (Ankom Technology Corporation Fairport, New York, USA). Neutral detergent fiber (NDF; inclusive of residual ash) was analyzed using ANKOM 200 Fiber Analyzer (Ankom Technology Corporation Fairport) following the NFTA (20) method, which includes alpha-amylase. Acid detergent fiber (ADF; inclusive of residual ash) was extracted sequentially after NDF analysis following method No. 1.9 A described in AFIA (21). Lignin was determined using ANKOM Technology method No. 9 by treating the ADF residues with sulphuric acid in a Daisy^II^ incubator (Ankom Technology Corporation Fairport). Soluble sugars were extracted using an ethanol: water (80:20) solution and quantified by colorimetric method using anthrone (22). The starch was hydrolyzed using enzymes and glucose was quantified by colorimetric method (AOAC 933.02). The mineral profile was analyzed for all the pooled samples (including pasture herbage) after nitric acid/peroxide digestion followed by inductively coupled plasma optical emission spectroscopy for all the minerals, except for chloride, which was quantified by potentiometric titration (23). The metabolizable energy concentration of STA, FIB concentrates and lucerne pellets was estimated by Hills Laboratory following method No. 7 from AFIA (21) after *in vitro* pepsin-cellulase DM digestibility determination following method No. 1.7R (AFIA) (21). The chemical composition of the individual ingredients of the diet is detailed in Table 1.

**Table 1:** Mean (± standard deviation) chemical composition of herbage, herbage silage (with mineral mix) and high starch (STA), high fiber (FIB) and lucerne concentrate pellets fed during the study with late lactation dairy cows.

Item	Herbage^a^	Herbage silage^b^	STA concentrate^c^	FIB concentrate^c^	Lucerne pellets^c^
DM, g/kg fresh matter	177 ± 21	384 ± 61	884 ± 11	895 ± 9	891 ± 19
Metabolizable energy, MJ/kg DM	11.5 ± 0.41	10.5 ± 0.58	11.8 ± 0.0	9.3 ± 0.07	10.0 ± 0.07
Macro profile, g/kg DM					
Ash	134 ± 0.97	149 ± 28.0	47 ± 0.7	61 ± 0.0	94 ± 0.7
Crude protein (CP)	216 ± 1.72	149 ± 11.8	160 ± 1.4	163 ± 0.7	130 ± 0.2
Crude fat	53 ± 0.37	41 ± 7.3	25 ± 1.4	38 ± 0.7	16 ± 0.7
Starch	nd	nd	375 ± 13.4	68 ± 0.7	77 ± 5.6
Soluble sugars	75 ± 1.03	84 ± 7.7	64 ± 0.7	58 ± 7.0	63 ± 8.4
Non-fiber carbohydrates (NFC)	166 ± 3.82	242 ± 15.3	499 ± 6.3	184 ± 0.7	286 ± 17.6
Acid detergent fiber	248 ± 2.60	250 ± 11.0	112 ± 3.5	332 ± 4.2	286 ± 25.4
Neutral detergent fiber (NDF)	431 ± 4.19	420 ± 31.9	270 ± 7.1	556 ± 0.7	476 ± 19.8
Lignin	nd	30 ± 6.1	26 ± 0.7	62 ± 0.7	22 ± 1.4
Mineral profile, g/kg DM					
Phosphorus	4.2 ± 0.29	3.8 ± 0.33	5.7 ± 0.06	6.2 ± 0.05	5.9 ± 0.51
Potassium	36.2 ± 5.99	35.5 ± 5.62	1.0 ± 0.03	12.6 ± 0.72	15.4 ± 0.12
Sulfur	3.9 ± 0.56	2.5 ± 0.36	1.9 ± 0.01	2.1 ± 0.01	2.0 ± 0.02
Calcium	6.6 ± 0.29	22.7 ± 9.24	6.2 ± 1.16	8.0 ± 1.74	18.2 ± 0.84
Magnesium	2.5 ± 0.23	4.0 ± 1.05	2.6 ± 0.07	3.5 ± 0.02	5.7 ± 0.2
Sodium	2.2 ± 0.62	4.8 ± 1.85	0.2 ± 0.01	0.1 ± 0.01	2.7 ± 0.94
Chloride	19.0 ± 2.60	16.6 ± 3.21	1.8 ± 0.01	2.0 ± 0.01	5.6 ± 0.11
DCAD, mEq/kg DM	242± 78	493± 157	97 ± 0.0	144± 18	231± 36

^a^Ryegrass-based herbage, chemical composition measured in individual samples using Near Infrared Spectroscopy, mineral composition analyzed in pooled samples using wet chemistry analyses (n = 3). ^b^Herbage silage mixed with mineral supplements. Mineral and organic composition measured with wet chemistry methods (n = 4). ^c^Chemical composition measured with wet chemistry methods (n = 2). NFC: calculated as 100 – (CP + ash + crude fat + NDF). DCAD: dietary cation-anion difference, calculated as [(sodium × 435) + (potassium × 256)] – [(chloride × 282) + (sulfur × 624)]. nd, non-determined.

### 2.4 Body weight, body condition score and animal behavior

The BW was recorded after morning milking for three consecutive days at the beginning and the end of the measurement phase using a static scale (X300, Tru-Test Datamars, Lugano, Switzerland). The three initial BW records and the three final records were averaged, and each averaged BW was considered as the initial and final BW, respectively. The body condition was scored at the beginning and the end of the measurement phase using a 10-point scale (24) by a single accredited scorer.

The cows' feeding behavior was recorded using Nedap SmartTags (Nedap Livestock Management, Groenlo, The Netherlands). The cow's behavior was classified into eating (biting and mastication without regurgitation), rumination (mastication with regurgitation), inactive (e.g., lying and not ruminating, or standing without walking/eating/ruminating) and other activity (remaining time not spent eating, ruminating or interactive, e.g., walking, drinking). The categorization of behaviors by the Nedap SmartTags was previously validated, including for grazing cows (25, 26).

A spreadsheet containing 31,572 unique 2-h interval records with the four activities per cow per day was generated by Nedap (Groenlo, The Netherlands). Data from the three calendar days when 32 cows went into respiration chambers were removed from the spreadsheet. In addition, rows where the data did not total 120 min were removed. On average, there were 413 ± 22 2-h interval records for each cow during the measurement phase. The minutes spent on each activity were averaged within each 2-h interval across the 36 measurement days. The minutes spent on each activity were then summed across the 12 2-h intervals in a 24-h day to generate daily minutes spent on each activity. The daily eating and rumination rates were calculated as the daily eating or rumination time divided by the total predicted DMI. Chewing time was calculated as the sum of daily time spent eating and ruminating.

### 2.5 Milk production and composition

Daily milk production (as L/d) was recorded automatically by the in-line milk meters of the milking system (Waikato Miking System). The milk production from the calendar days of those 32 cows that went into respiration chambers was not included in the dataset (i.e., four milkings). During the measurement phase, a subsample of milk was collected weekly during the morning and afternoon milkings. These milk samples were stored at 4 °C for less than 24 h until milk composition analysis. Milk fat, protein, casein, lactose, and urea concentrations were determined in successive morning and afternoon samples using a Milkoscan (FT1, Foss Electric, Hillerød, Denmark) at Te Rourou (BSI, Palmerston North, New Zealand).

To estimate the production of each milk component per milking, the concentration of each milk component from milk samples (g/100 g milk) was obtained once a week for morning and afternoon milkings. The concentration of each component at each milking was multiplied by the weekly average milk production for morning and afternoon milking, respectively. The daily production of each milk component (kg/d), calculated at a weekly resolution, resulted from the sum of the morning and afternoon production of each component. The weekly data was averaged across the measurement weeks and the actual concentration of each milk component reported in the results section (g/100 g milk) was calculated by dividing the daily production of each component (kg/d) by the daily milk production (kg/d) and multiplying by 100. Total milk solids (TS) production was calculated as the sum of protein and fat production per day. The fat and protein-corrected milk production (FPCM; corrected to 4% fat and 3.3% protein) was calculated as: = [0.337 + (0.116 × % fat) + (0.06 × % protein)] × milk production (kg) (27). Milk density measured from each sample was used to transform the volume of milk into the mass of milk.

### 2.6 Dry matter intake estimation

The total DMI was estimated weekly and then averaged across weeks. The DMI of STA, FIB and lucerne pellets were measured as described above. The intake of herbage silage was assumed to be the same amount for all cows. The total DM of silage offered per day was divided by the number of cows at the paddock every day, and the silage utilization efficiency was assumed to be 60% based on crude visual observations of the refusals. The DMI of pasture herbage was assumed to be equal to the total metabolizable energy requirements (MEr; MJ/d) of individual cows minus ME supplied by DMI from concentrates (including lucerne pellets) and herbage silage and then dividing the remaining MEr by the ME content (MJ/kg DM) of pasture herbage. The total MEr was calculated from the ME requirements for maintenance (MEm), grazing (MEg), lactation (MEl), gestation (MEc) and body weight change (MEwt) according to equations from CSIRO (18). Input parameters for the MEr equations are age (years), BW (kg), BCS, daily milk production, milk composition, live weight change and days pregnant.

### 2.7 Methane and carbon dioxide estimates: data processing

Sixty-five cows out of the initial 72 cows (90% of the cows) visited the GF units at least once during the measurement phase. Five out of the 65 cows that visited the GF during the measurement phase were removed during the experiment due to mastitis or lameness (one from CTR, one from MIX, one from FIB and two from STA), resulting in 60 experimental units (CTR: *n* = 15, MIX: *n* = 16, FIB: *n* = 14; STA: *n* = 15).

Methane production from each spot sample was plotted against visit duration to visually examine data dispersion, following an approach similar to Bennett et al. (28). The variation in CH_4_ output observed for visits lasting 1.5–2 min was comparable to that seen during visits of 2 min or longer. As a result, visits within the 1.5–2.0-min range were retained in the dataset. The number and timing of visits to the GF units were unequal between animals, in each hour of the day and between days. To take this into account, the production of gases per animal was estimated using a linear mixed model that included GF unit, day within animal and hour in the day within the day as random effects to predict daily emissions of CH_4_ and CO_2_ (g/d). The data of the 60 cows that had visited the GF during the measurement phase were included in the model. The gas emissions for each cow were calculated with the “estimate_grouplevel” function of the “modelbased” package (29) in R software (30) to extract random parameters of each individual in the context of mixed models.

The residual CH_4_ was estimated as the residual error in CH_4_ emissions of animal *i* from fitting a linear regression between CH_4_ emissions as a function of CO_2_ emissions.

### 2.8 Statistical analysis

The first exclusion criterion was failure to visit the GF units. Of the 72 cows initially enrolled, 65 visited the GF units and seven did not. Four of the seven cows that failed to visit were removed due to health issues preventing visitation. Among the 65 cows that visited the GF units, five were subsequently excluded because health issues affected longitudinal data collection of the variables of interest.

The eating behavior might be related to the GF unit visitation patterns; thus, the effect of dietary treatments was tested on the eating and rumination times, including 72 cows or only cows that visited the GF units. Eating and rumination time was analyzed using a linear mixed model that included the dietary treatment as a fixed effect and parity block (two, three, and >three) as a random effect. The eating time was affected by dietary treatment when including 72 cows (*P* < 0.01), while the rumination time was not (*P* = 0.52). Similar results were found using 60 cows (included in the final analyses) for eating (*P* < 0.01) and rumination (*P* = 0.75). Therefore, although 72 cows (*n* = 18 per treatment) were included in the experiment, statistical analyses were performed using only those 60 cows that visited the GF units and had all the other variables measured over time.

The analysis of the data from the measurement phase was performed using the “predictmeans” and “lme4” packages in the statistical software R version 4.3 (30). Data from 60 cows (CTR: *n* = 15, MIX: *n* = 16, FIB: *n* = 14; STA: *n* = 15; cow served as the experimental unit) were analyzed with a statistical model that included the dietary treatment as a fixed effect and parity block (two, three, and >three) as a random effect. For the milk production-related variables as well as CH_4_ emissions per unit of FPCM (CH_4_/FPCM) and per unit of TS (CH_4_/TS), production worth index (i.e., breeding value) was included as a covariate in the statistical model. The rumination and eating time data in 2-h intervals were analyzed using a repeated measurements model that included dietary treatment, time interval (i.e., 12 2-h intervals) and their interaction as fixed effects, and cow ID nested within parity block as a random effect. The variance-covariance matrix was unstructured, meaning it places no constraints on variances or covariances, allowing each time point and pair of repeated measurements to be estimated freely [“lme4” package (31)]. This approach avoids imposing a specific correlation pattern but requires sufficient data to support reliable parameter estimation.

For each model, the normality and homoscedasticity of the errors were evaluated by plotting the residuals against the predicted values. A multiple treatment comparison was performed on the modeling results with *P*-value adjusted by the “BH” method (32). The analyses were performed using the “predictmeans” package (33). The significance of mean differences was declared at *P* < 0.05. Trends were considered when *P*-values were >0.05 and < 0.10. Pearson correlations were performed between CH_4_ emission-related variables and cow's behavior parameters.

## 3 Results

### 3.1 Diet composition, gas emissions, and milk production

The initial and final BW and BCS were similar across the dietary treatments (Table 2). Estimated DMI was greater in FIB cows compared to CTR fed cows, while MIX and STA-fed cows had an intermediate DMI (*P* < 0.01). Concentrate intake expressed as proportion of DMI was greater in STA and MIX-fed cows compared to FIB cows (*P* < 0.01). The concentrate feeding decreased the dietary concentrations (g/kg DMI) of crude fat (*P* < 0.01) and crude protein (*P* < 0.01; CTR>FIB> MIX> STA), while increasing the concentration of non-fiber carbohydrates (NFC; *P* < 0.01) in the reverse order. The concentrations of NDF (*P* < 0.01) and ADF (*P* < 0.01) in the diet were greater in FIB fed cows followed by CTR, MIX and STA-fed cows.

**Table 2:** Body weight, intake, number of visits to the GreenFeed units, methane (CH_4_) and carbon dioxide (CO_2_) emissions, and feeding behavior in grazing late lactation dairy cows without supplementation (CTR) or supplemented with high starch (STA), high fiber (FIB) or mixed (50:50) (MIX) concentrates.

Item	Dietary treatment				SED	*P*- value
	CTR (*n* = 15)	FIB (*n* = 14)	MIX (*n* = 16)	STA (*n* = 15)		
Body mass/condition						
Initial body weight, kg	533	528	522	537	19.6	0.87
Final body weight, kg	531	533	525	539	19.5	0.92
Initial body condition score^1^	4.2	4.3	4.4	4.2	0.18	0.78
Final body condition score^1^	4.4	4.5	4.4	4.2	0.13	0.87
Dry matter and nutrient intake						
DMI, kg/d^2^	16.0^b^	18.4^a^	17.7^ab^	17.3^ab^	0.68	< 0.01
Concentrate/DMI, %	0^c^	26^b^	29^a^	30^a^	1.1	< 0.01
Ryegrass herbage/DMI, %	89^a^	64^b^	62^bc^	59^c^	1.3	< 0.01
Herbage silage/DMI, %	7.4	6.4	6.7	6.9	0.29	0.08
Lucerne pellets/DMI, %	3.4	2.8	2.7	3.2	0.48	0.73
Ash, g/kg DMI	133^a^	114^b^	110^c^	106^d^	0.9	< 0.01
Crude protein, g/kg DMI	207^a^	194^b^	192^bc^	191^c^	0.9	< 0.01
Crude fat, g/kg DMI	51^a^	47^b^	45^c^	42^d^	0.4	< 0.01
NDF, g/kg DMI	435^b^	467^a^	429^c^	385^d^	1.5	< 0.01
ADF, g/kg DMI	251^b^	273^a^	243^c^	209^d^	1.1	< 0.01
NFC, g/kg DMI	174^c^	178^c^	224^b^	275^a^	3.0	< 0.01
GreenFeed visits						
Total visits/cow	82	78	81	85	12.4	0.95
Visits cow/d	2.5	2.1	2.5	2.3	0.33	0.61
Duration/visit, min	3.13	3.09	3.01	3.05	0.090	0.52
Gas emissions						
CH_4_, g/d	353.8	342.5	349.3	362.9	14.28	0.56
CO_2_, g/d	11,984	11,779	11,834	12,084	324.4	0.78
CH_4_, g/kg DMI	21.8^a^	18.4^b^	19.6^ab^	20.8^ab^	1.12	0.02
CH_4_/CO_2_, mol/mol	0.08	0.08	0.08	0.08	0.002	0.96
CH_4_/FPCM, g/kg^3^	24.0^a^	21.5^ab^	20.7^b^	20.4^b^	1.25	0.02
CH_4_/TS, g/kg^3^	311.5	293.7	275.4	272.2	19.4	0.15
Residual CH_4_	−0.29	12.58	1.92	6.25	10.36	0.63
Cow behaviors						
Eating time, min/d	618^a^	567^b^	582^b^	572^b^	10.3	< 0.01
Rumination time, min/d	434	450	448	439	16.7	0.75
Chewing time, min/d	1,052	1,016	1,029	1,011	19.0	0.15
Inactive time, min/d	362	396	388	399	18.9	0.20
Other activities, min/d	26	28	21	30	5.75	0.43
Eating rate, min/kg DMI	39.2^a^	31.1^b^	33.4^b^	33.7^b^	1.72	< 0.01
Rumination rate, min/kg DMI	27.5	24.6	25.6	25.9	1.49	0.29

Table contains the least square means. CTR: 0 kg/d of concentrate, STA: 5 kg/d of high starch concentrate, FIB: 5 kg/d of high fiber concentrate, MIX: 2.5 kg/d of high starch concentrate + 2.5 kg/d of high fiber concentrate, SED, average standard error of the difference; DMI, dry matter intake; NFC, non-fiber carbohydrates calculated as 1,000 – (ash + NDF, crude protein + crude fat), NDF, neutral detergent fiber; ADF, acid detergent fiber; FPCM, fat and protein-corrected milk; TS, calculated as protein plus fat production. ^a − d^Means with different letters within a row are different at *P* < 0.05. ^1^Body condition score at a 10 point scale. ^2^Estimated based on metabolizable energy requirements. ^3^P-value for production worth used as a covariate for CH_4_/FPCM and CH_4_/TS are 0.235 and 0.596, respectively.

**Figure 1:**
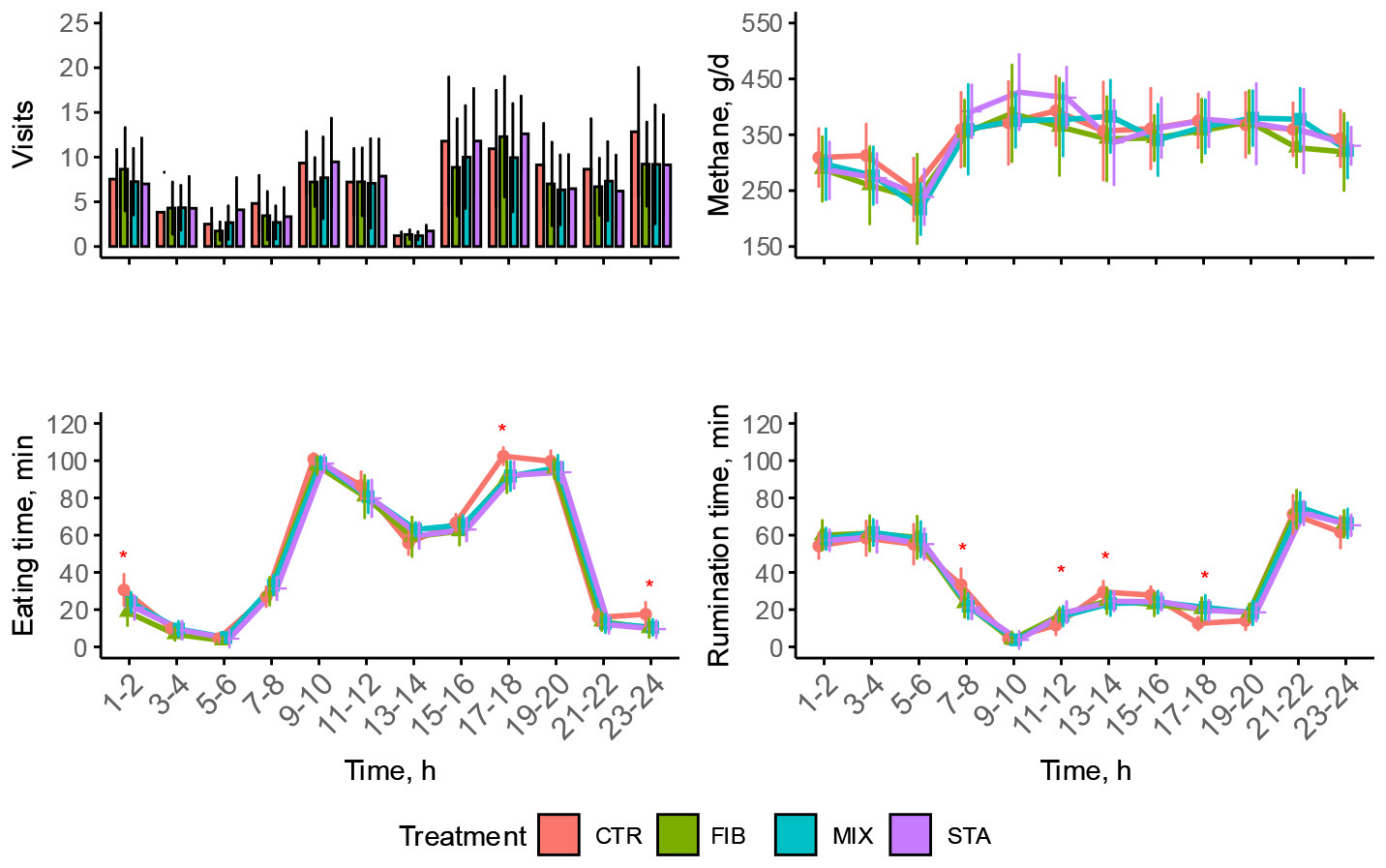
Mean and standard deviation (whiskers) of accumulated number of visits to GreenFeed units **(top left)**, methane production **(top right)**, eating **(bottom left)** and rumination **(bottom right)** time in grazing late lactation dairy cows without supplementation (CTR) or supplemented with high starch (STA), high fiber (FIB) or mixed (MIX; 50: 50) concentrates. Dietary treatment × hour interaction: *P* < 0.01 for eating and rumination time. *Indicates means among dietary treatment differ within a 2-h interval at *P* ≤ 0.05.

The total number of visits to the GF units per cow, the average visits per cow per day and the average visit duration were similar among the four dietary treatments. The cows did not have access to the GF units between ~06:15 to 07:45 h and ~13:15 to 14:45 h while being away from the paddock for milking, which explains the low number of visits to the GF during those time intervals (Figure 1).

The CH_4_ production followed a circadian pattern, decreasing steadily from midnight until cows had access to a new strip of pasture after morning milking (Figure 1). After that, the CH_4_ production increased and stayed relatively stable until midnight. The CH_4_ and CO_2_ emissions, as well as the CH_4_ to CO_2_ ratio were similar between the four dietary treatments. The CH_4_ yield was 15% less in FIB-fed cows compared to CTR cows (*P* = 0.02), while STA and MIX-fed cows had an intermediate CH_4_ yield. The CH_4_/FPCM was different between dietary treatments (*P* = 0.02), while the CH_4_/TS was similar between dietary treatments (*P* = 0.15). The CH_4_/FPCM was 14 and 13% less in MIX and STA compared to CTR-fed cows, with FIB-fed cows intermediate.

The FPCM was 16% greater in MIX and STA compared to CTR cows (*P* < 0.01), with FIB cows intermediate (Table 3). The TS production was greater (*P* = 0.026) in STA than in CTR and FIB cows, with MIX intermediate. The protein, casein and lactose yields (kg/d) were less in CTR than in STA and MIX-fed cows, and less in FIB than in STA fed cows (*P* = 0.003). The proportions of milk constituents analyzed were similar among cows of the four dietary treatments. Milk urea tended to be greater in CTR than in FIB (*P* = 0.07).

**Table 3:** Milk production and composition in grazing late lactation dairy cows without supplementation (CTR) or supplemented with high starch (STA), high fiber (FIB) or mixed (50:50) (MIX) concentrates.

Item	Dietary treatment				SED	***P*** **-value**	
Item	CTR (*n* = 15)	FIB (*n* = 14)	MIX (*n* = 16)	STA (*n* = 15)		Cov	Treatment
Milk composition, g/100 g milk							
Fat	5.56	5.52	5.13	5.18	0.25	0.91	0.18
Protein	4.20	4.26	4.24	4.16	0.10	0.34	0.77
Casein	3.23	3.28	3.27	3.20	0.08	0.32	0.71
Lactose	4.67	4.62	4.66	4.69	0.04	0.34	0.36
Urea, mg/100 g milk	27.4	23.9	26.9	26.5	1.33	0.56	0.07
Milk production parameters, kg/d							
Milk	11.8^c^	12.2^bc^	13.9^ab^	15.6^a^	0.76	0.75	< 0.01
FPCM	14.9^b^	16.1^ab^	17.1^a^	18.0^a^	0.80	0.19	< 0.01
TS	1.15^b^	1.18^b^	1.28^ab^	1.34^a^	0.07	0.52	0.03
Fat	0.66	0.66	0.70	0.74	0.04	0.60	0.17
Protein	0.50^c^	0.52^bc^	0.58^ab^	0.60^a^	0.03	0.45	< 0.01
Casein	0.38^c^	0.40^bc^	0.45^ab^	0.46^a^	0.02	0.43	< 0.01
Lactose	0.55^c^	0.56^bc^	0.65^ab^	0.68^a^	0.04	0.66	< 0.01

Table contains the least square means. SED: average standard error of the difference; Cov: production worth covariate. CTR: 0 kg/d of concentrate, STA: 5 kg/d of high starch concentrate, FIB: 5 kg/d of high fiber concentrate, MIX: 2.5 kg/d of high starch concentrate + 2.5 kg/d of high fiber concentrate. FPCM, fat and protein-corrected milk; TS, calculated as protein + fat production. ^a − c^Means with different letters within a row are different, *P* < 0.05.

### 3.2 Cow behaviors

The eating time (min/d) was greater in CTR cows compared to supplemented cows (*P* < 0.01), while the daily rumination time was similar among cows of the four dietary treatments (*P* = 0.75; Table 2). The daily chewing time (rumination plus eating time), daily inactive time, daily time dedicated to other activities, and daily rumination rate were similar among cows of the four dietary treatments. The daily eating rate (min/kg DMI) was 16% less in supplemented cows compared to CTR cows (*P* < 0.01). Daily eating and rumination rate as min/kg DMI had a moderate positive correlation with CH_4_/FPCM and CH_4_/TS (*r* = 0.30–0.44; Table 4). Daily eating and rumination rates (min/kg DMI) were highly correlated to each other (*r* = 0.69). Daily eating time (min/d) was moderately correlated with predicted DMI (*r* = −0.24), while rumination time was not (*r* = 0.04). Residual CH_4_ was positively correlated with CH_4_ emissions (g/d; *r* = 0.35) and CH_4_/FPCM (*r* = 0.23).

**Table 4:** Pearson correlation coefficients between feeding behavior parameters and methane emissions parameters in late lactation grazing dairy cows without supplementation or supplemented with high starch, high fiber or mixed (50:50) concentrates.

Item	DMI, kg/d	CH_4_, g/d	CH_4_, g/kg FPCM	CH_4_, g/kg TS	Residual CH_4_, g/d
Ruminating, min/d	0.04	0.13	−0.05	0.02	0.10
Eating, min/d	−0.24^δ^	0.01	0.22^δ^	0.19	−0.16
Chewing, min/d	−0.13	0.11	0.10	0.14	−0.02
Inactive, min/d	0.15	−0.07	−0.05	−0.08	0.04
Other activities, min/d	−0.07	−0.17	−0.17	−0.23^δ^	−0.05
Rumination rate, min/kg DMI	nd	−0.01	0.30^*^	0.31^*^	0.10
Eating rate, min/kg DMI	nd	−0.10	0.44^**^	0.39^**^	−0.03
Residual methane, g/d	−0.01	0.35^*^	0.23^δ^	0.16	nd

^**^P < 0.01; ^*^P ≤ 0.05; ^δ^0.05 < *P* < 0.10. DMI, dry matter intake; CH_4_, methane; FPCM, fat and protein corrected milk; TS, calculated as protein plus fat production; nd, non-determined.

The time spent ruminating (min/2 h) was affected by the dietary treatment and time of the day (*P* < 0.01; Figure 1). Across all dietary treatments, rumination activity increased around 20:00 h and was maintained until 06:00 h, while rumination activity was less from 10:00 h until dusk (around 20:00 h). The rumination activity was similar among dietary treatments during most of the 2-h intervals, except from 07:00 to 08:00 h and from 13:00 to 14:00 h when the CTR cows ruminated for longer than supplemented cows, and from 11:00 to 12:00 h and from 17:00 to 18:00 h when CTR cows ruminated less than supplemented cows.

The time spent eating (time within 2-h interval) was also affected by the dietary treatment and the time of the day (*P* < 0.01). Across all dietary treatments, the eating activity (min/2 h) was the lowest from dusk (from 18:00 h) to dawn (until 06:00 h), highest after each milking from 08:00 to 12:00 and from 16:00 to 20:00 h, and intermediate from 12:00 to 16:00 h. Within 2-h intervals, the time spent eating was similar among the four dietary treatments, except from 23:00 to 02:00 h and from 17:00 to 18:00 h. During those specific time intervals, the CTR cows spent more time eating, resulting in 20% more-time eating compared to supplemented cows in 24 h (*P* < 0.01).

## 4 Discussion

The supplementation with any of the three concentrates did not affect the CH_4_ production significantly but increased the FPCM compared to CTR cows, and consequently resulted in a decreased CH_4_/FPCM for supplemented cows (i.e., dilution effect). The FPCM production increased with STA and MIX concentrates compared to CTR, but to a different extent, with an intermediate FPCM production for FIB.

### 4.1 Effect of concentrate feeding on milk production and methane emissions

In the current experiment, yields of milk, FPCM, TS, protein, casein and lactose consistently followed the order CTR < FIB < MIX < STA. The FPCM production response (relative to CTR) per kg concentrate DM was approximately 0.24, 0.44, and 0.62 kg/d for FIB, MIX and STA, respectively. Predicted DMI was 1–2 kg greater for cows fed concentrates compared to CTR cows and starch and NFC increased with changing concentrate type in the order FIB < MIX < STA, which might explain the observed response in milk yield parameters. Walker et al. (34) and Bargo et al. (6) also indicated that the FPCM response was generally greater with starch-rich concentrates than with high fiber concentrates in grazing cows. The increase in FPCM production in grazing cows in response to concentrate supplementation is generally driven by an increment in the total DMI and/or a greater supply of energy, glucogenic precursors and/or protein (6). Predicted DMI was 1–2 kg greater for cows fed concentrates compared to CTR cows and starch and NFC increased with changing concentrate type in the order FIB < MIX < STA, which might explain the observed response in milk yield parameters. Grazing early lactation cows supplemented with 0, 2, 4, and 6 kg of concentrate pellets also had a linear increase in FPCM production, due to increased DMI and energy supply, leading to a linear decrease in CH_4_/FPCM (10, 35). A decrease in CH_4_/FCPM will only lead to a decreased absolute CH_4_ production at the farm level if FPCM production remains constant (i.e., reduce number of cows to produce the same amount of FPCM).

The CH_4_ production was similar among treatments in the current study and therefore CH_4_/FPCM was less for MIX and STA cows compared to CTR cows as their FPCM production was greater (i.e., dilution effect). The CH_4_ production was also observed to remain similar in the earlier study with the same cows fed graded levels of concentrates (34) and in the meta-analysis of Arndt et al. (9). To our knowledge, no other study has directly compared the effect of feeding high starch and high fiber concentrates in grazing dairy cows on CH_4_ emissions, however, several studies compared these concentrate types with other basal diets. Hatew et al. (36) and Pirondini et al. (37) fed high and low starch concentrates (change in corn to fibrous by-product ratio in both studies) in lactating dairy cows and found no difference in FPCM production or CH_4_ emissions expressed as g/d, g/kg DMI, or g/kg FPCM. Hindrichsen et al. (38) fed six concentrate types with different main carbohydrate type (e.g., starch, inulin, pectin, sucrose, fiber; including from oat hulls and soybean hulls and high starch wheat) in the diet of lactating cows at a level to meet their energy requirements and found no difference in CH_4_ production and intensity expressed as g/kg milk protein but observed a lower CH_4_ yield for cows fed oat hulls than the other treatments including wheat. Multiple-regression analysis with their data suggested that the lower CH_4_ yield due to oat hulls was due to the indigestible lignified nature of the oat hulls. The findings in these three studies largely align with similar milk production parameters and gas emissions in cows fed the three concentrate treatments in the current study. Different to these findings, Bougouin et al. (39) found CH_4_ emissions (g/d, g/kg DMI and g/kg FPCM) to be less in cows fed starch rich than in cows fed fiber rich concentrates, while FPCM production was similar between treatments.

The CH_4_ production of CTR cows in the current study (354 g/d) was less than previously found in mid to late lactation grazing cows in New Zealand (384–402 g/d) (40), but similar to that observed in the same cows during early lactation in spring receiving no concentrates (350 g/d; 10) and grazing late lactation dairy cows in Ireland (352 g/d) (41). While Jonker et al. (40) reported a higher DMI (17.9 kg/d) compared to the predicted DMI for the CTR cows in the current study, Starsmore et al. (41) reported an average DMI (16.6 kg/d) which was closer to the DMI predicted for the CTR cows. Furthermore, the variations in CH_4_ production between studies evaluating non-supplemented grazing cows may be related to the quality of the herbage (13) and to the DMI level. Increments in the DMI increase the digesta passage rate (42) and a faster turnover in the rumen is associated with less CH_4_ production (43).

### 4.2 Relationship between CH_4_ emissions and feeding behavior parameters

Slower eaters and slower ruminators (more time spent per kg of DMI) showed higher CH_4_ production per unit FPCM. This relationship appeared to be associated with lower FPCM production in these animals rather than an increase in absolute CH_4_ production. Behavioral traits and CH_4_ intensity may be jointly influenced by underlying factors such as intake, diet selection, and rumen fermentation dynamics. In a grazing system, herbage eaten becomes of lower quality over time as the sward is grazed down from the top to the bottom (44, 45), and therefore, slower grazers might on average have consumed lower quality pasture, which would lead to lower FPCM production.

A greater eating and rumination rate has been previously associated with a longer digesta retention time in the digestive tract and enhanced feed digestibility (42). A faster digesta passage rate, potentially associated with lower fiber fermentation but compensated by higher DMI, could contribute to the similar CH_4_ production among cows fed herbage only or herbage plus supplements.

Daily eating time had a weak relationship with the estimated DMI, as was previously found in cattle fed a lucerne silage in respiration chambers (12). Pasture-related factors such as pasture structure, grazing pressure and botanical composition add complexity to the use of the eating time as a predictor of CH_4_ production in grazing cattle (46, 47). Similarly, the daily rumination time was found to be poorly correlated with DMI in the current study and also by Zetouni et al. (48).

Regardless of the dietary treatment, all the cows followed a similar rumination and eating pattern driven by pasture access, daylight times and circadian rhythms. Most of the rumination activity occurred after dusk and before dawn, while most of the eating activity occurred from dawn to dusk, which was similar to that observed in previous studies with grazing lactating dairy cows in New Zealand (49, 50). The eating activity increased after the morning milking, likely reflecting access to a new pasture strip, whereas rumination increased during non-eating periods.

The supplemented cows spent less time on the eating activity compared to CTR cows, likely because they received 5 kg DM/d from concentrates and ate this in less than 30 min (i.e., daily milking time). The eating time was similar for cows on the different concentrate treatments. Although the rumination activity from CTR cows was less at some time intervals and greater at other time intervals compared to supplemented cows, the 24 h sum of time spend ruminating was similar. The lack of differences in total daily rumination time among dietary treatments may reflect the high proportion of herbage in all dietary treatments. Overall, eating time appeared more responsive to dietary changes than rumination time, as also reported by Jiang et al. (51).

### 4.3 Limitations of this study

As for any *in situ* grazing study, DMI cannot be measured directly; therefore, intake was predicted from productive variables. Although this approach applies common parameters across animals and may not fully capture all inter-animal variation, individual performance data are the main drivers of the estimates and account for much of this variability. Data from previous studies indicate that accurate CH_4_ yields can be generated when combining measure CH_4_ production with predicted DMI, but with a lower precision and greater uncertainty (52, 53). The GF system for measuring CH_4_ emissions relies on voluntary visitation of cows and there was no access to the units around milking. As a consequence of low visitation, only 60 out of 72 cows had sufficient CH_4_ data, which reduced the statistical power of the study (54).

## 5 Conclusion

High starch or mixed concentrates could be used as a supplement during summer to increase the milk production from grazing late lactation dairy cows and decrease the CH_4_ production per unit of FPCM, but not to decrease the CH_4_ production per cow. Rumination time was more stable to changes in the diet of grazing cows without or with concentrate supplementation than eating time.

## Data Availability

The raw data supporting the conclusions of this article will be made available by the authors, without undue reservation.
